# Editorial: Wiring the young mind: neural correlates of language development in early childhood

**DOI:** 10.3389/fnint.2026.1941106

**Published:** 2026-08-07

**Authors:** Avantika Mathur, Yingying Wang

**Affiliations:** 1 Department of Psychology and Human Development Peabody College, Vanderbilt University, Nashville, TN, United States; 2 Special Education and Communication Disorders, Center for Brain, Biology and Behavior, University of Nebraska-Lincoln, Lincoln, NE, United States

**Keywords:** bilingualism and functional networks, developmental neuroimaging, early language development, language risk markers, social context and early learning

Language acquisition represents one of the most remarkable achievements of early human development, and modern neuroscience is advancing our understanding of the neural mechanisms supporting this complex process. Despite decades of behavioral research documenting critical periods for language learning and the importance of early environmental input, significant questions persist about how brain structure and function translate into linguistic competence. Moreover, identifying neural markers of language risk in early childhood remains a substantial clinical challenge, limiting our ability to intervene during windows of maximal plasticity.

This collection of articles explores the neural foundations of early language development through four complementary lenses: the impact of prenatal environmental exposures on white matter development and language outcomes, the role of early bilingual experience in shaping functional brain organization for cognitive control, the role of social interaction in shaping brain-behavior relationships, and the utility of neurophysiological measures for predicting language ability in at-risk populations. Together, these contributions illustrate how modern neuroimaging techniques—from diffusion MRI to magnetoencephalography (MEG) to functional near-infrared spectroscopy (fNIRS)—are revealing the complex interplay between environmental factors, neural development, and linguistic competence in the critical early years.

## 1 Environmental risk and neural pathways to language

A foundational question in developmental neuroscience concerns how prenatal environmental exposures shape the neural substrates of cognition. Scholten et al. address this question directly by examining white matter microstructure and language skills in South African toddlers with prenatal tobacco and/or alcohol exposure. Using diffusion tensor imaging (DTI) in 93 children aged 2–3 years, they found that while exposed and unexposed children showed comparable language scores, prenatal substance exposure (PSE) significantly moderated the relationship between white matter microstructure and early language skills.

Specifically, children with PSE demonstrated lower mean diffusivity in the splenium of the corpus callosum compared to controls—a finding that persisted after correction for multiple comparisons. More intriguingly, PSE altered the brain-language relationship across seven white matter tracts, though these interactions did not survive correction. The exposed group showed more positive associations between fractional anisotropy and language scores and more negative associations between mean diffusivity and language scores compared to controls, suggesting that PSE may alter how white matter properties support linguistic processing.

These findings are particularly important because they demonstrate neural differences in the absence of behavioral deficits in Expressive or Receptive language scores, suggesting that neuroimaging may detect subtle alterations in developmental trajectories before they manifest in overt delays. The authors propose that altered white matter development in PSE children may reflect premature maturation patterns or compensatory reorganization that could influence later language outcomes. This work underscores the value of neuroimaging for identifying children who may benefit from early intervention, even when current behavioral assessments appear unremarkable.

## 2 Experiential factors and functional networks

Cook et al. examine how early bilingual experience shapes prefrontal functional organization during cognitive control. Using functional near-infrared spectroscopy (fNIRS) during an interference suppression task, they compared 13 bilingual and 13 monolingual preschoolers and found no behavioral differences in accuracy or reaction time. However, bilingual children exhibited more localized prefrontal connectivity patterns with reduced activation in key decision-making regions, suggesting greater neural efficiency. This demonstrates how early language experience restructures functional brain networks in ways not captured by behavioral performance alone—the constant practice of language switching may refine executive control circuits even when behavioral advantages are not yet evident.

## 3 Social foundations: from behavioral to neural synchrony

While structural brain imaging reveals how prenatal factors shape the neural architecture available for language learning, understanding language acquisition also requires examining the social contexts in which learning occurs. Eulau and Hirsh-Pasek provide a comprehensive review synthesizing decades of behavioral research with emerging neuroimaging findings to illuminate how caregiver-child interaction shapes linguistic and cognitive development.

The authors trace a conceptual arc from early work on joint attention and coordinated joint engagement through contemporary research on neural synchrony measured with hyperscanning techniques. They detail how socially contingent interactions—characterized by both semantic relevance and temporal contiguity—create optimal learning environments for young children. Importantly, the timing of caregiver responses matters: infants show enhanced brain responses to verbal input delivered within 300–400 milliseconds, and caregiver responsiveness within approximately 2 s supports symbol-referent mapping. For instance, when an infant picks up or gazes at an object, the caregiver's timely labeling of that object within this critical window allows the infant to map the symbol to the object while it remains the focus of their multisensory attention.

The review's most significant contribution lies in synthesizing behavioral and neural evidence to show that social contingency may influence development beyond language. Dual-recording fNIRS studies demonstrate that caregiver-child neural synchrony during interaction predicts not only language outcomes but also attention, emotion regulation, and problem-solving abilities. For instance, studies show that infants whose caregivers track their attentional focus more precisely show enhanced sustained attention and that preschoolers solving problems cooperatively with caregivers show increased prefrontal synchrony associated with better task performance.

Crucially, Eulau and Hirsh-Pasek argue that neural synchrony measures add substantial value beyond behavioral data by: (1) capturing interaction dynamics at sub-second timescales, (2) revealing which specific behaviors activate or deactivate during interaction, (3) providing truly dyadic rather than individual-level measures, and (4) illuminating interdependencies between seemingly separate constructs like attention and language. This framework suggests that the “socially-gated” brain may support not just language acquisition but broader cognitive scaffolding through contingent social interaction.

## 4 Neural markers of language risk

If neuroimaging can detect subtle alterations in children exposed to prenatal risks and reveal mechanisms through which social interaction supports language, can it also identify neural markers that predict individual differences in language ability? Chen et al. demonstrate that it can, using MEG to examine auditory processing in preschoolers with elevated likelihood of intellectual disability or developmental delay (ID/DD-EL).

Their study enrolled 30 three- and 4-year-old children with ID/DD-EL and used a passive auditory task to measure the M50 response—an early cortical evoked response reflecting auditory encoding speed, yielding evaluable data in 23 children. They discovered that left hemisphere M50 latency significantly predicted language ability: longer latencies (slower auditory encoding) were associated with lower language scores. Notably, M50 latency predicted language specifically, not general cognitive ability, suggesting domain-specific relevance.

The left-hemisphere specificity is particularly noteworthy. The authors note that the left auditory cortex matures more slowly than the right throughout development, and this extended maturation may be particularly sensitive to factors affecting language development. Their findings extend earlier work in older children with autism and minimal verbal abilities, demonstrating that auditory cortex response latencies can predict language outcomes even in very young children at risk for developmental delays.

Importantly, Chen et al. achieved these findings using a passive auditory task requiring no active participation—an approach that can succeed across a wide range of developmental levels and cognitive abilities. Their 77% success rate in obtaining evaluable MEG data from 3-year-olds with developmental concerns, using their MEG-PLAN protocol, demonstrates the feasibility of neuroimaging in challenging populations. This accessibility is crucial for translating neuroscience findings into clinical tools that can inform early intervention.

## 5 Converging themes and future directions

Together, these contributions illuminate three converging themes that define the current landscape of developmental language neuroscience.

### 5.1 Neural measures reveal risk and mechanism before behavioral deficits emerge

The three empirical studies each demonstrate dissociations between neural and behavioral measures, and the Eulau and Hirsh-Pasek review synthesizes further evidence of such dissociations from the broader literature. Scholten et al. found altered brain-language relationships in PSE children despite comparable language scores. Cook et al. revealed that bilingual and monolingual preschoolers showed identical behavioral performance on an interference suppression task, yet bilingual children exhibited significantly more localized prefrontal connectivity patterns with reduced global connections, suggesting greater neural efficiency in managing cognitive control demands. Chen et al. identified M50 latency as a predictor of individual differences within an ID/DD-EL sample in which most children fall within the normal range of cognitive and language ability. These findings suggest that neuroimaging can detect vulnerability, experience-driven reorganization, or compensatory mechanisms that precede or exist alongside equivalent behavioral performance, opening windows for understanding developmental plasticity and informing preventive intervention.

### 5.2 Multiple neuroimaging modalities provide complementary insights

The articles collectively employ DTI, MEG, and fNIRS directly, with EEG-based hyperscanning findings discussed in the review—each offering distinct advantages. DTI reveals structural connectivity that may constrain or enable function. MEG provides millisecond-resolution measurement of neural activity essential for understanding auditory processing. Task-based fNIRS enables measurement of functional connectivity patterns during active cognitive tasks in young children, capturing how brain regions coordinate during real-time processing. Dual-recording fNIRS and EEG discussed in the review enable measurement of interactive, dyadic processes that cannot be captured by individual brain recordings. The field's maturation is reflected in researchers' strategic selection of modalities matched to specific questions rather than reliance on any single technique.

### 5.3 Social and biological factors interact to shape neural development for language

The reviewed work moves beyond simplistic nature-versus-nurture frameworks. Eulau and Hirsh-Pasek's review demonstrates how social interaction shapes neural development through contingent, precisely-timed exchanges. Scholten et al. show how prenatal environmental factors alter structural brain development, potentially changing how children extract information from their social environment. Cook et al. illustrate how early experiential factors—specifically, dual language exposure—reshape functional brain organization for cognitive control, demonstrating that language experience itself serves as a powerful environmental sculptor of neural architecture. Chen et al. demonstrate individual differences in neural processing that predict language outcomes even in the context of known genetic and biological risk factors. Together, these works suggest that effective intervention will require addressing biological vulnerabilities, experiential factors, and social-environmental supports as interacting components of a developmental system.

## 6 Challenges and opportunities

Despite substantial progress, significant challenges remain. First, sample sizes in developmental neuroimaging studies remain relatively small, limiting statistical power and generalizability. Cook et al.'s sample of 26 participants (13 per group) and Chen et al.'s evaluable sample of 23 (of 30 enrolled) participants, while comparable to similar studies, illustrate this constraint. Collaborative, multi-site studies and data-sharing initiatives will be essential for building adequately powered datasets.

Second, the field must address issues of accessibility and representativeness. All four articles acknowledge limitations related to socioeconomic factors and note their efforts to control for these confounds. However, neuroimaging research remains concentrated in well-resourced settings, and most samples are not representative of global diversity. The Scholten et al. study of South African children represents valuable progress toward more inclusive research, but much more work is needed. Additionally, as Cook et al. note, bilingualism research must account for the substantial heterogeneity within bilingual populations—including language pairs, age of acquisition, proficiency levels, and cultural contexts—factors that likely interact with neural outcomes.

Third, translating neuroimaging findings into clinical tools requires additional validation. While Chen et al.'s M50 latency findings are promising, prospective longitudinal studies are needed to determine whether this marker predicts later language outcomes and whether interventions can modify both the neural measure and behavioral trajectory. Similarly, the functional connectivity patterns identified by Cook et al. require longitudinal investigation to determine whether early neural efficiency translates into sustained cognitive or linguistic advantages across development.

Finally, as Eulau and Hirsh-Pasek emphasize, the field must move toward integrated, multi-measure approaches. The field of developmental language neuroscience would benefit from standardized protocols, shared analysis pipelines, and integration of multiple neural measures with rich behavioral and environmental data.

